# Increased Incidence of *Candida auris* Colonization in Early COVID-19 Pandemic, Orange County, California, USA

**DOI:** 10.3201/eid3109.241342

**Published:** 2025-09

**Authors:** Alissa H. Dratch, Mi Le, Matthew Zahn

**Affiliations:** Orange County Health Care Agency, Santa Ana, California, USA (A.H. Dratch, M. Le, M. Zahn)

**Keywords:** *Candida auris*, COVID-19, fungus, cumulative incidence, competing risks, survival analysis, healthcare-associated infections, LTACH, point prevalence surveys, admission screening, pandemic, California, United States

## Abstract

*Candida auris* transmission surged in long-term acute-care hospitals (LTACHs) in Orange County, California, USA, during the COVID-19 pandemic. This study describes the effect of COVID-19 on *C. auris* transmission by estimating the probability of patient colonization in LTACHs across 5 epidemiologic time periods. Patients had the highest probability of developing new skin colonization during the first COVID-19 wave, with a cumulative incidence of 22.5% (95% CI 18.5­–26.6) after a 30-day stay. Once the initial COVID-19 waves abated, a reduction in cumulative incidence of *C. auris* colonization was observed concurrently with persistent high prevalence, indicating that within-facility transmission can be reduced with proper infection prevention and control practices. Admission screenings and point prevalence surveys provided a wealth of data that guided public health recommendations and supported the objectives of both public health professionals and LTACHs for monitoring facility transmission dynamics and guiding decision making.

*Candida auris* is a multidrug-resistant yeast that can cause serious invasive infections in at-risk populations (*1*) and an emerging pathogen in the United States that can cause outbreaks in healthcare settings. Persons with extensive healthcare exposure, indwelling medical devices, or recent antimicrobial drug use are at highest risk for *C. auris* colonization or infection (*2*,*3*). Because of the high prevalence of patients with those risk factors, *C. auris* is frequently found in long-term acute-care hospital (LTACH) settings (*4*,*5*). *C. auris* is a reportable pathogen in Orange County, California, USA and all known cases are reported to the Orange County Healthcare Agency (OCHCA).

The first outbreak in California was identified in Orange County after *C. auris* was detected in a urine specimen from an LTACH patient in February 2019. Local, state, and federal public health agencies mounted an aggressive containment response, which included active surveillance, to identify transmission in 3 LTACHs and 14 ventilator-equipped skilled nursing facilities serving adult patients in Orange County (*6*,*7*). Because of their high-risk patient populations, the 3 LTACHs in the county agreed to perform routine point prevalence surveys (PPSs) that consisted of screening all noncolonized patients at their facilities via axilla and groin swab sampling. The first PPSs at each LTACH were performed in March 2019. During March 2019–September 2020, PPS frequency varied with transmission patterns. PPSs were generally done 1–2 times per month at each LTACH, though there was variance because they were largely done in response to identification of new cases. In September 2020, the LTACHs switched to routine PPS schedules. From then on, PPSs routinely occurred 1–2 times per month regardless of the level of *C. auris* activity at individual facilities.

By November 2020, in response to widespread transmission, all 3 LTACHs implemented universal admission screening of patients not known to be *C. auris* positive. That testing enabled the rapid identification of colonized patients and early implementation of infection prevention precautions. In addition, the strategy created the opportunity to track patients longitudinally starting from their admission date. Thus, OCHCA was able to collect individual-level longitudinal screening data over 5 epidemiologically distinct time periods, which corresponded with 1 pre–COVID-19 period and 4 periods during the COVID-19 pandemic. OCHCA was able to track *C. auris* spread as the fungus became endemic to the county and while the county’s facilities were simultaneously responding to multiple COVID-19 surges. This study was conducted to objectively assess whether the suspected patterns of colonization rates across different phases of the pandemic in Orange County were supported by empirical data and to gain insights to inform future infection prevention and response activities.

## Methods

**Figure 1:**
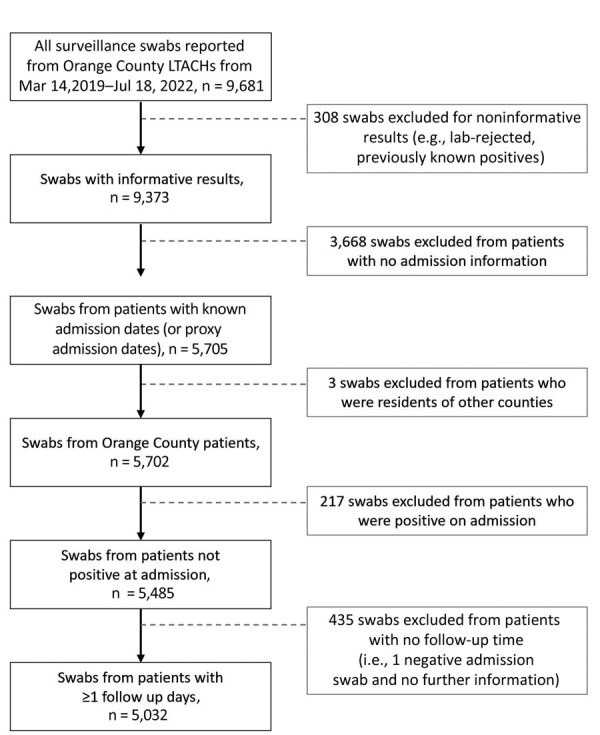
Flowchart showing cohort swab specimen collection and testing in study of *Candida auris* colonization early in the COVID-19 pandemic, Orange County, California, USA. LTACH, long-term acute-care hospital.

This cohort study examined results from all axilla and groin surveillance swab specimens from the 3 LTACHs in Orange County during March 14, 2019–July 18, 2022 (Figure 1). We planned the study shortly after the implementation of universal admission screening, so data from November 2020 on were collected prospectively. Because of OCHCA’s close working relationships with the LTACHs, we received data as the test results became available. We validated, cleaned, and prepared data for analysis on an ongoing basis as we received test results.

During sample collection, a single swab was used to swab both axilla and both inguinal creases. We tested the swabs from PPSs through the Centers for Disease Control and Prevention’s Antimicrobial Resistance Laboratory Network or through Orange County’s Public Health Laboratory by using PCR testing methods. If an indeterminate result was returned, the same swab specimen that was already tested was sent for culture. Admission swab specimens were tested through the LTACHs’ private laboratory, where they used culture methods only.

Axilla and groin screening swab specimens that tested positive for *C. auris* indicated skin colonization, and we counted them as cases. In addition, 3 patients who had clinical cultures (such as blood or bronchoalveolar lavage samples) positive for *C. auris* before the yeast was detected via PPS screening were counted as cases. Those patients were considered positive on the collection date of the positive clinical sample. Once a patient was counted as a case, that patient was permanently considered a case and would not be rescreened.

**Figure 2:**
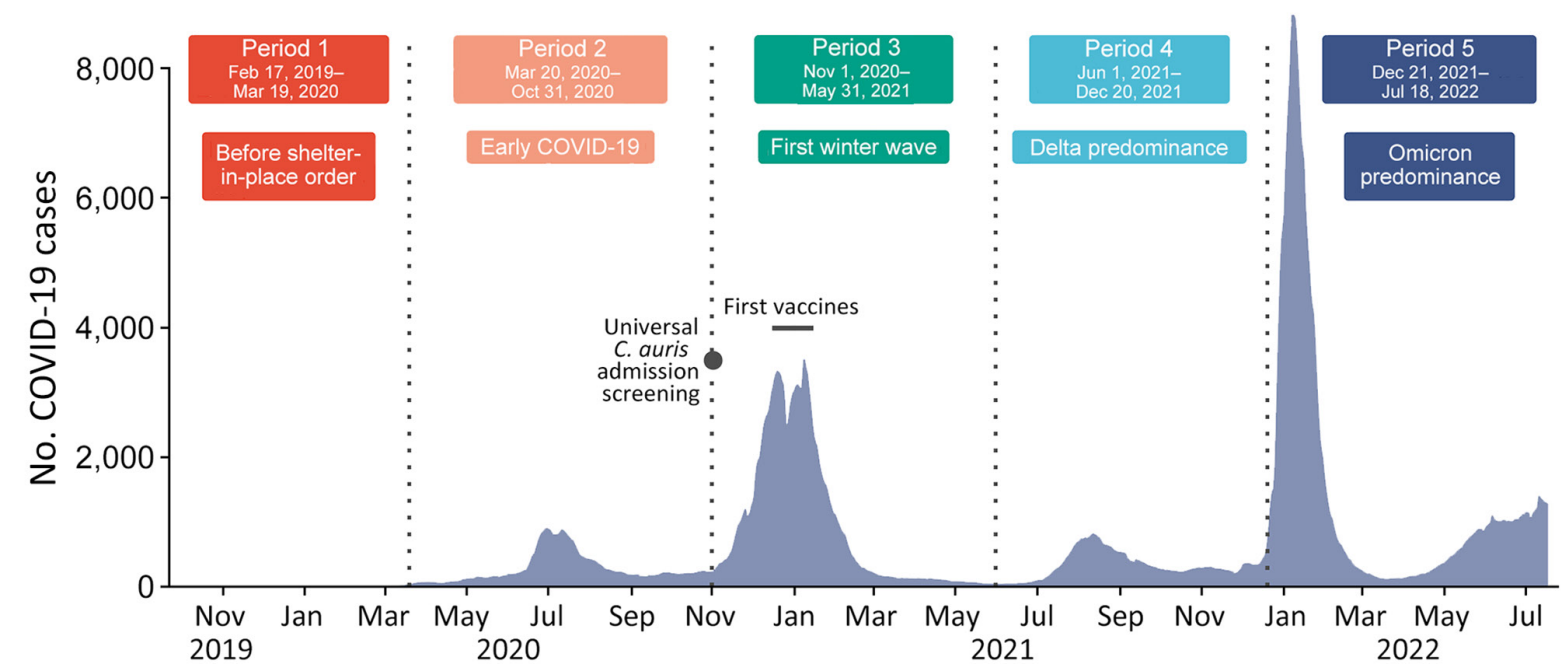
Seven-day moving averages of new daily COVID-19 cases in Orange County, California, USA, across 5 time periods during study of *Candida auris* colonization early in the COVID-19 pandemic. Black dot represents beginning of *C. auris* screening. Horizontal black bar represents period in which COVID vaccines first became available.

The study duration was divided into 5 time periods for closer examination: 1 period before the initial COVID-19 shelter-in-place order and 4 periods during the COVID-19 pandemic (Figure 2). The period cut points were determined a priori on the basis of OCHCA’s understanding of local COVID-19 and *C. auris* epidemiology. The cut points largely reflect COVID-19 surges and the corresponding fluctuations in infection prevention and control (IPC) resource availability. In instances where patients’ exposure time straddled 2 time periods (6.4% of swab specimens), the time was assigned to the period in which most of the days were spent. We conducted a sensitivity analysis that excluded follow-up time that straddled multiple time periods, and it showed negligible effect on the results.

We ascertained death dates from Orange County’s vital records data. Because the records included location of death and were well populated, we were able to verify patients were still in LTACHs on their date of death.

### About the LTACHs

The average censuses among the 3 LTACHs during the 2019–2022 study period ranged from 74% to 83% of the licensed bed number, according to data from the Office of Statewide Health Planning and Development Annual Utilization Report of Hospitals (*8*,*9*,*10*,*11*). The largest facility had 109 licensed beds, and the smaller facilities had 48 and 54 licensed beds. Most rooms in the facilities served as multioccupancy rooms (typically 2–4 patients); however, if there was an infection prevention need and facility capacity, some rooms could be configured into single-occupancy rooms.

### Exposure Time Calculation

We counted exposure days as the number of cumulative days a patient was admitted to an OC LTACH without a positive *C. auris* test. Exposure days were usually, but not necessarily, continuous. A patient could have exposure time from multiple admissions not temporally connected to each other.

We counted exposure time beginning at a patient’s admission date (or admission screening date as proxy). For patients admitted to LTACHs before *C. auris* was first detected in the county, we imputed a start date of February 17, 2019, which is the collection date of the earliest detected *C. auris* case in Orange County. Exposure time stopped accumulating on the date of a patient’s last swab or when a patient died. The 3 possible outcomes were a patient received a negative *C. auris* test and was then discharged and lost to follow-up, a patient received a positive *C. auris* test, or a patient died.

### Sampling Differences During Periods 1 and 2

To avoid left-censoring, we excluded 3,668 swabs belonging to 1,732 patients, almost entirely from time periods 1 and 2, before universal admission surveillance was implemented. No admission dates were recorded for those patients, so we could not determine when their exposure time began.

### Statistical Analysis

We plotted the cumulative incidence function estimating the probability of *C. auris* skin colonization for up to 45 days of exposure time during each period and accounted for patient death as a competing risk. Our primary outcome of interest was a difference in subdistribution estimates of *C. auris* skin colonization across time periods. We conducted Gray’s test to test for equality of cause-specific cumulative incidence functions for each pair of time periods (e.g., periods 1 and 2, periods 1 and 3, periods 1 and 4) (*12*). We then adjusted the p values for multiple comparisons by using Holm’s method (*13*). Next, we extracted point estimates and 95% CIs from the cumulative incidence curves at 30 days of exposure time for descriptive analysis. We chose 30 days because it was the average patient stay length in an LTACH (*14*). We overlaid and compared the extracted estimates with median *C. auris* prevalence rates from PPSs at Orange County LTACHs during each period.

To assess the effect of the smaller sample sizes during periods 1 and 2, we conducted a second sensitivity analysis in which we imputed estimated admission dates for the left-censored patients on the basis of our knowledge of past PPS dates. We plotted cumulative incidence curves and extracted point estimates and 95% CIs at 30 days of exposure time. We did not choose this approach for the final analysis because of an inability to exclude patients who were positive for *C. auris* before LTACH admission. Although the percentage of patients positive at admission was expected to be low during periods 1 and 2, particularly because admission screening was not universal, the limited availability of admission screening data prevented us from drawing that conclusion. Only 218 (7.5%) patients tested positive at admission during the full study period (0 in period 1, 6 [3.9%] in period 2, 51 [6.6%] in period 3, 78 [8.0%] in period 4, and 83 [8.3%] in period 5). We did not conduct significance testing. We computed statistics and generated plots by using the ggsurvfit (*15*), tidycmprsk (*16*), and cmprsk (*17*) packages in R version 4.3.0 (The R Project for Statistical Computing, https://www.r-project.org).

## Results

The analysis included 5,032 screening swab specimens from 1,935 patients, totaling 45,343 days of associated exposure time. We ascertained an additional 43 patient-days from 3 clinical cases and 4,511 patient-days from death data. We identified 307 total *C. auris* cases for inclusion (Table 1). Patients had median of 17 (interquartile range 8–30) days of follow up time and a median of 2 (interquartile range 2–3) surveillance swab specimens.

**Table 1:** *Candida auris* colonization frequency counts in 3 LTACHs, by time period of cases, surveillance swabs, and exposure time, during early COVID-19 pandemic, Orange County, California, USA*

Characteristic	Period					Total
	Before shelter-in-place order	Early COVID-19	First winter wave	Delta predominance	Omicron predominance	
	1	2	3	4	5	
Cases						
Skin colonization	9	15	154	57	72	304
Clinical case	0	0	3	0	0	3
Surveillance swab specimens						
Total	355	367	1,658	1,425	1,227	5,032
At admission	16	145	674	703	628	2,166
Exposure time						
Total patient-days	4,624	2,870	15,426	13,044	13,927	49,891

*Axilla and groin surveillance swab specimens were collected from patients in the 3 LTACHs in Orange County during March 14, 2019–July 18, 2022. Date ranges for each study period are shown in Figure 2. LTACH, long-term acute-care hospital.

**Figure 3:**
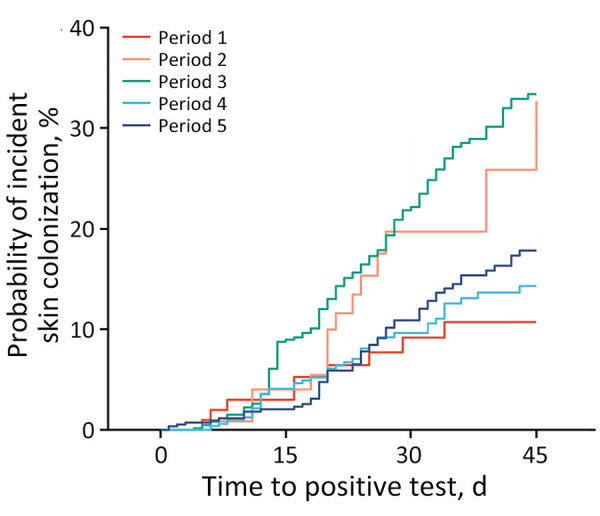
Cumulative incidence of *Candida auris* after 45 days of long-term acute care hospital exposure across 5 periods in study of *C. auris* colonization early in the COVID-19 pandemic, Orange County, California, USA. Date ranges for each study period are shown in Figure 2.

The plotted cumulative incidence functions for the 5 time periods show markedly different trajectories (Table 2; Figure 3). After adjustment for multiple comparisons, we found the curve for period 3 to be significantly different from periods 1, 4, and 5 (each p<0.001) (Table 3). Patients in OC LTACHs during period 3 had a higher probability of skin colonization than patients in periods 1, 4, or 5. The curve for period 2 appears most visually similar to period 3, but because of the small sample size during period 2, we could not detect significant differences between period 2 and other time periods.

**Table 2:** Number of patients at risk for *Candida auris* colonization early in the COVID-19 pandemic in 3 LTACHs, by study period, Orange County, California, USA*

Study period	No. patients at 0 days	No. patients at 15 days	No. patients at 30 days	No. patients at 45 days
Period 1	104	84	53	40
Period 2	145	65	21	9
Period 3	692	364	172	81
Period 4	612	295	128	68
Period 5	563	325	159	86

*Cohort consisted of patients in the 3 LTACHs in Orange County during March 14, 2019–July 18, 2022. Date ranges for each study period are shown in Figure 2. LTACH, long-term acute-care hospital.

**Table 3:** Comparison of *Candida auris* colonization rates in 3 LTACHs, by study period, during early COVID-19 pandemic, Orange County, California, USA*

Period comparison	Unadjusted Gray's test	Adjusted for multiple comparisons
1 and 2	0.032	0.224
1 and 3	**<0.001**	**<0.001**
1 and 4	0.115	0.557
1 and 5	0.038	0.229
2 and 3	0.171	0.557
2 and 4	0.111	0.557
2 and 5	0.207	0.557
3 and 4	**<0.001**	**<0.001**
3 and 5	**<0.001**	**<0.001**
4 and 5	0.423	0.557

*Bold values represent significance at p = 0.05. Cohort consisted of patients in the 3 LTACHs in Orange County during March 14, 2019–July 18, 2022. Date ranges for each study period are shown in Figure 2. LTACH, long-term acute-care hospital.

Patients in periods 2 and 3 had the highest probabilities of skin colonization developing after 30 days of exposure; point estimates were 19.7% (95% CI 10.7–30.7%) of patients for period 2 and 22.5% (95% CI 18.5–26.6%) of patients for period 3 (Table 4). Patients in periods 1, 4, and 5 had lower probabilities: 9.2% (95% CI 4.2–16.5%) of patients for period 1, 9.6% (95% CI 6.8–13.0%) for period 4, and 10.9% (95% CI 7.9–14.5%) for period 5. The sensitivity analysis with imputed admission dates yielded similar estimates but with smaller CIs.

**Table 4:** Probability of *Candida auris* skin colonization in 3 LTACHs, by time period and cumulative exposure time, during early COVID-19 pandemic, Orange County, California, USA*

Time period	Cumulative exposure time		
	15 days	30 days	45 days
1	3.0% (0.8%–7.8%)	9.2% (4.2%–16.5%)	10.7% (5.2%–18.6%)
2	4.0% (1.3%–9.3%)	19.7% (10.7%–30.7%)	32.7% (15.1%–51.6%)
3	9.0% (6.7%–11.6%)	22.5% (18.5%–26.6%)	33.4% (28.3%–38.5%)
4	4.1% (2.5%–6.2%)	9.6% (6.8%–13.0%)	14.3% (10.4%–18.8%)
5	2.0% (1.0%–3.6%)	10.9% (7.9%–14.5%)	17.8% (13.6%–22.5%)

*Values are point estimates (95% CI). Cohort consisted of patients in the 3 LTACHs in Orange County during March 14, 2019–July 18, 2022. Date ranges for each study period are shown in Figure 2. LTACH, long-term acute-care hospital.

**Figure 4:**
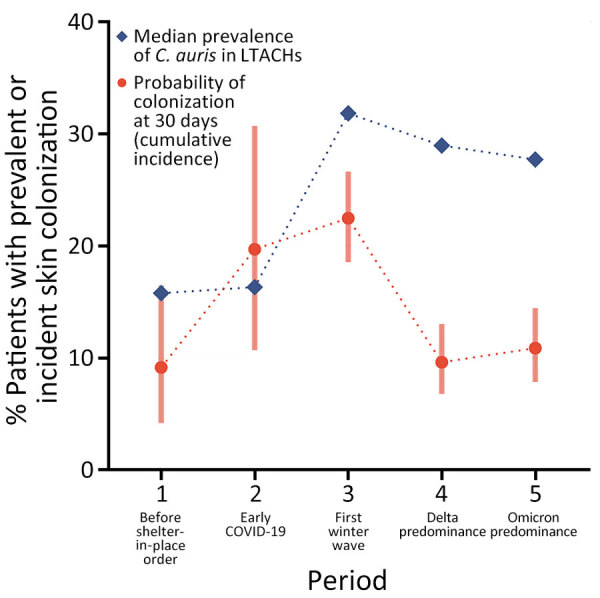
Point estimates for the cumulative incidence and median prevalence of *Candida auris* skin colonization in long-term acute care hospitals across 5 periods in study of *C. auris* colonization early in the COVID-19 pandemic, Orange County, California, USA. Date ranges for each study period are shown in Figure 2. LTACH, long-term acute-care hospital.

The probability of skin colonization occurring after 30 days of exposure rose between periods 1 and 3 and then dropped to be similar to prepandemic levels in periods 4 and 5 (Figure 4). The median *C. auris* prevalence rates from PPSs in LTACHs did not mirror the pattern we saw with cumulative incidence. Instead, median prevalence rates rose dramatically from periods 1 to 3, then remained high during periods 4 and 5.

## Discussion

Patients admitted to LTACHs in Orange County had a substantial risk of becoming colonized with *C. auris* as the pathogen emerged in the community. The risk was most pronounced during the earliest phase of the COVID-19 pandemic and during the first winter wave of COVID-19 cases (periods 2 and 3), emphasizing how pandemic-related logistical challenges faced by healthcare facilities played a pivotal role in *C. auris* transmission dynamics. Similar to other facilities nationwide, LTACHs in OC experienced critical operational challenges including shortages of personal protective equipment and disruptions to IPC practices, exacerbated by widespread concern over COVID-19 infection among both staff and patients (*18*,*19*). In response to the public health emergency, resources typically allocated to preventing the spread of pathogens such as *C. auris* were redirected to COVID-19 prevention efforts (*20*). Specifically, patient cohorting protocols prioritized COVID-19 status over *C. auris* colonization status (*21*). All those factors might have contributed to the sharp rise in *C. auris* skin colonization observed during periods 2 and 3.

After the initial COVID-19 surges abated and vaccines became more freely available, probability of *C. auris* skin colonization after 30 days of LTACH exposure decreased to approximately baseline prepandemic levels (during periods 4 and 5). The decrease occurred despite higher community prevalence of *C. auris* and the appearance of Delta- and Omicron-related COVID-19 waves. The combination of higher prevalence but lower cumulative incidence seen during those periods indicates that within-facility transmission can be reduced with close adherence to IPC. However, although transmission was mitigated, it was not eliminated. Newly colonized case-patients continued to be identified routinely across all time periods. This result is consistent with findings from previous studies showing that, once *C. auris* transmission is established in LTACHs, preventing ongoing transmission becomes exceptionally challenging because of long-term patient colonization and persistence of *C. auris* on nosocomial surfaces (*22*,*23*).

The wealth of data collected from PPSs helped guide public health recommendations and enabled OCHCA to track transmission dynamics as *C. auris* transitioned from emerging to established transmission in OC. That type of patient-level longitudinal data is highly valuable but rarely available, especially in real-time. Building infrastructure that makes data collection and use more accessible could evolve our understanding of pathogen transmission dynamics and improve public health guidance for intervention and response efforts.

As of March 2025, all 3 local LTACHs in Orange County continue to perform admission screenings through their private laboratories. OCHCA continues to support PPSs in those facilities, albeit at a more relaxed interval of approximately every 3 months. Both measures require a major resource investment from LTACHs and public health; however, both entities recognize the value and importance of those efforts. Investing in prevention can save on resources used to respond to outbreaks and can prevent potentially serious infections in at-risk patients. Future modeling efforts could distinguish the individual effects of both admission screenings and PPSs on *C. auris* transmission. By identifying the most effective applications of each approach, those models could help optimize resource use and reduce the overall investment required.

Our analysis likely missed cases in all time periods. Skin colonization has been documented to occur in as little as 4 hours after exposure (*24*); it is possible that some patients were exposed after admission but before their admission swab specimen was collected and were mistakenly counted as positive at admission and excluded from the model. Similarly, some patients may have had skin colonization develop shortly before discharge or death, and those would not always be captured. In addition, although the surveillance tests rarely produce false-positive results, they have imperfect sensitivity and can potentially produce false-negative results because of several factors (*25*–*27*).

The exclusion of swab specimens from patients without admission dates introduced sampling bias for periods 1 and 2. During those periods, patients who tested positive or who were exposed to a known *C. auris* case (i.e., had a positive roommate or resided in a room previously occupied by a positive patient) had more complete data collection because of OCHCA conducting individual-level case investigations when *C. auris* was new to Orange County. Of consequence, those patients were more likely to have recorded admission dates and be included in the analysis, potentially biasing the estimates upwards. This fact was especially true during period 2, when our resources were diverted to COVID-19 response and contact tracing efforts for *C. auris* were downsized, creating further bias as to which patients received screenings. We handled the possible bias by taking it into consideration during interpretation of the results. We understood the probability of skin colonization to be very low during period 1 when *C. auris* was new to Orange County and believe it is likely overestimated in this study. Therefore, the finding that period 1 had a lower rate of skin colonization compared with period 3 should be considered conservative. In addition, the results of the sensitivity analysis with imputed admission dates indicate that the estimates for periods 1 and 2 are stable despite the smaller sample sizes. We expected the cumulative incidence to remain overestimated in the sensitivity analysis because PPSs were performed in response to identification of new cases during those periods, namely, when the chances of identifying new cases was highest. This understanding, combined with the smaller sample size, again makes statements about period 1 estimates being lower than those from other periods conservative.

This study is insufficient to evaluate the public health effect of PPSs and universal admission screening. To do so, we would need a model that could account for changes in the underlying patient population and transmission dynamics over time, particularly considering changes related to the first several COVID-19 surges. The model in this study does not delineate the individual contribution of admission screenings, PPSs, or other critical factors.

Our findings contribute to existing literature by quantifying the effect of IPC disruptions on *C. auris* transmission during the early stages of the COVID-19 pandemic and by highlighting the substantial risk of colonization once *C. auris* is introduced into an LTACH. Data from PPSs and admission screenings can serve as a valuable tool for monitoring facility transmission dynamics and for guiding decision making. Enhanced data collection and modeling might further clarify the role of admission screenings and PPSs in reducing the spread of *C. auris* and other hospital-acquired infections.
